# Reduction of Silver Ions by Cell Free Extracts of *Westiellopsis* sp.

**DOI:** 10.1155/2015/539494

**Published:** 2015-02-01

**Authors:** P. T. V. Lakshmi, Devi Priyanka, A. Annamalai

**Affiliations:** ^1^Department of Bioinformatics, Bharathiar University, Coimbatore 641 046, India; ^2^Phytomatics Laboratory, Centre for Bioinformatics, School of Life Sciences, Pondicherry University, Puducherry 605 014, India; ^3^Plant Molecular Biology Laboratory, Department of Biotechnology, School of Biotechnology and Health Sciences, Karunya University, Coimbatore 641 114, India

## Abstract

Biosynthesis of silver nanoparticles using *Westiellopsis* sp. (A15), a filamentous cyanobacterium belonging to the family Fischerellaceae, has been demonstrated. Aqueous silver ions (Ag^+^) when exposed to the culture filtrate of *Westiellopsis* were reduced in the solution, which were characterized by biophysical measures utilizing the UV-Vis spectroscopy, scanning electron microscopy (SEM), and FTIR. The nanoparticles exhibited the maximum absorbance at 420 nm in UV-Vis spectroscopy, while the SEM micrograph revealed that the aggregated nanoparticles vary in size between 20 nm and 5 *µ*m. However, the FTIR analysis provided evidence for presence of proteins in the filtrate to be involved in the reduction of silver ions.

## 1. Introduction

An important area of research in nanotechnology deals with the synthesis of nanoparticles of different chemical compositions, dimension, and controlled monodispersity. Currently, there is a growing need to develop environmentally benign nanoparticle synthetic processes that are free from toxic chemicals in the synthesis protocol and, as a result, researchers have turned to biological systems employing either microorganisms or plant extracts as a simple and viable alternative to chemical and physical methods. There are many different types of inorganic nanoparticles being synthesized from the biological sources, of which the synthesis of silver nanoparticles has attracted much attention because of their unique shape-dependent optical, electrical, chemical, and antibacterial properties that are widely applied in photographic reactions, catalysis, chemical analysis [1], and prevention of diseases [2]. The first report of live plants synthesizing nanoparticles [3] especially gold nanoparticles (ranging in size from 2 to 20 nm) from alfalfa seedlings and subsequently the synthesis of spherical silver nanoparticles from the same plant when exposed to a silver rich solid medium [4] led to the discovery of characterization of extracellularly and intracellularly synthesized silver nanoparticles in many other microbes such as* Pseudomonas stutzeri* AG259 [5],* Escherichia coli*,* Staphylococcus aureus* [6],* Phoma* sp. 3.2883 [7] and* Vibrio cholerae*,* Pseudomonas aeruginosa,* and* Salmonella typhus* [8].

Although many proofs for synthesizing silver nanoparticle from various biological sources have been reported, not much is being investigated on the cyanobacterial forms which are one of the largest and most important groups of photoautotrophic bacteria on earth that are widely applied in all the fields including biodegradation and bioaccumulation process [9, 10]. Hence an attempt was made in this direction to screen for the formation of silver nanoparticles in the filamentous cyanobacterium,* Westiellopsis* sp. (A15) of Fischeralleceae.

## 2. Materials and Methods

### 2.1. Chemicals

Silver nitrate (AgNO_3_) as analytical grade, citric acid, ferric ammonium citrate, sodium nitrite, magnesium sulphate, calcium chloride, sodium carbonate, EDTA disodium salt, zinc sulphate, manganese chloride, copper sulphate, sodium molybdate, and cobalt nitrate were purchased from Himedia Laboratories Pvt., Ltd., while dipotassium hydrogen phosphate and boric acid were purchased from SRL (Mumbai, India).

### 2.2. Culture Maintenance

Culture of* Westiellopsis* sp. (A15) was obtained from the Culture Collection of Algae, Centre for Advanced Studies in Botany, University of Madras, Chennai, and was maintained in BG_11_ medium [11] with citric acid (0.006 g/L), ferric ammonium citrate (0.006 g/L), sodium nitrite (1.5 g/L), magnesium sulphate (0.075 g/L), calcium chloride (0.003 g/L), sodium carbonate (0.01 g/L), EDTA disodium salt (0.001 g/L), dipotassium hydrogen phosphate (0.040 g/L), and trace metals solution (1 mL) with the mixture of boric acid (2.86 g/L), zinc sulphate (0.222 g/L), manganese chloride (1.81 g/L), copper sulphate (0.079 g/L), sodium molybdate (0.390 g/L), and cobalt nitrate (0.494 g/L), respectively. The culture flasks were maintained in growth chamber (under 12/12 h light/dark cycle by fluorescent illumination of 40 *μ*Em^−2^s^−1^) fitted with Frontier digital timer at 27 ± 1°C. Gentle shaking of the cultures was done manually every day to reduce the clumping of cells.

### 2.3. Harvesting of Biomass

The culture was grown for approximately 4–6 weeks to reach the stationary growth phase and was harvested according to the protocol of Lengke et al. [9], where the cells were centrifuged at 8000 ×g for 5 min and washed with sterile distilled water followed by extensive washing with sterile double-distilled water to remove salts and trace metals from the medium before being used for the experiments.

### 2.4. Synthesis of Silver Nanoparticles

Typically, 10 g (wet weight of biomass) after thorough washing with sterile double-distilled water was brought into contact with 100 mL of sterile double-distilled water for 48 h at 27°C in an Erlenmeyer flask and agitated at 150 rpm on an orbital shaker (ORBITEK) as given by Maliszewska et al. [12]. After the incubation, the cell filtrate was filtered by Whatman filter paper number 1 wherein to 100 mL of cell filtrate a carefully weighed quantity of silver nitrate was added to yield an overall Ag^+^ ion concentration of 10^−3 ^M, and the reaction was carried out in the dark [13].

### 2.5. Characterization of Silver Nanoparticles

#### 2.5.1. UV-Vis Spectra Analysis

Surface plasmon resonance of silver nanoparticles was characterized using a UV-visible spectrophotometer (Shimadzu 3600 Spectrophotometer) at the resolution of 1 nm from 200 to 700 nm as given by Krishnaraj et al. [14]. After complete reduction of AgNO_3_ ions by the cell extract, the solution was centrifuged at 14,000 rpm for 30 minutes (SIGMA 3K30, GERMANY) to isolate Ag nanoparticle free from proteins or other bioorganic compounds present in solution. The Ag nanoparticles pellet obtained was redispersed in water and washed (centrifugation and redispersion) with distilled water for 2 times and then air-dried for characterization [15].

#### 2.5.2. SEM Analysis of Silver Nanoparticles

The air-dried silver nanoparticles were subjected to scanning electron microscope (FEI, Japan) which works on the basis of electron beam that typically has an energy range from few keV to 50 keV. The accelerating voltage of the microscope was kept in the range 10–20 kV. The samples were made on glass slides which were fixed on aluminium stubs and then allowed to get dry and subsequently a thin layer of gold was coated to customize the sample for conduction. The micrograph was recorded by focusing on clusters of particles [16].

#### 2.5.3. FTIR Spectra Analysis

The FTIR spectrum of the dried nanopowder was recorded by Nicolet 6700 Fourier transform infrared spectroscopy (Nicolet, USA). The sample was prepared by mixing 10% of the dried nanopowder with 90% of potassium bromide (KBr) pellet and was characterized with the spectrum ranging between 450 and 4000 cm^−1^ at a resolution of 4 cm^−1^ [17].

## 3. Results and Discussions

The biomimetic approaches in search of nanomaterials especially from the organisms that have the ability to remediate the toxic metals via reduction of the metal ions/formation of metal sulphides have provoked the material scientists to explore microorganisms such as bacteria and yeast to prologue as a possible ecofriendly nanofactories [18]. In the present study, an effort made of this kind is solely a new report because although many other forms of cyanobacteria have been investigated for the synthesis or production of different nanoparticles including silver, gold, and platinum, species of* Westiellopsis* have never been reported so far.* Westiellopsis* sp. (A15), investigated for the formation of silver nanoparticle, revealed a positive scope for extending research in this organism.

### 3.1. UV/Vis Spectrum Confirming the Formation of Silver Ions

**Figure 1:**
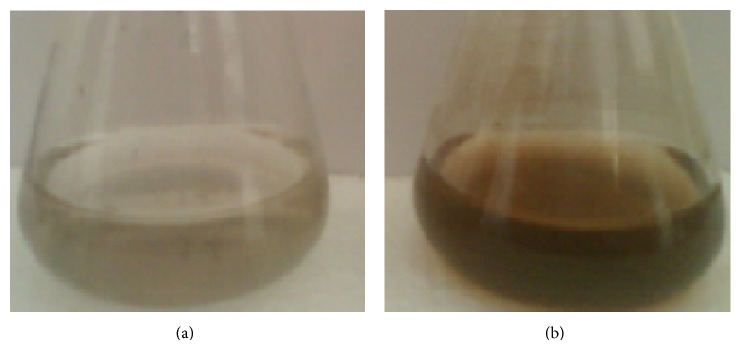
Optical photographs represent the synthesis of silver ions. (a) Control (cyanobacterial cell filtrate without treatment); (b) cyanobacterial cell filtrate treated with 1 mM silver nitrate.

Upon addition of Ag^+^ ions (1 mM AgNO_3_) into the filtered cell-free culture extract at 27°C in the dark, the sample challenged color change from almost colorless to light brown in 10 days and intensively increased with the period of incubation (next 10 days) indicating the formation of silver nanoparticle. Classically, the electric field of an incoming wave induces a polarization of the electrons with respect to much heavier ionic core of silver nanoparticles that results in a net charge difference, which in turn acts as a restoring force to create a dipolar oscillation of all the electrons with the same phase. When the frequency of the electromagnetic field becomes resonant with the coherent electron motion, a strong absorption takes place, which is the origin of the observed colour (brown) whose absorption strongly depended on the particle size, dielectric medium, and chemical surroundings [19, 20]. However, control (without silver ions) showed no color change of the cell filtrates when incubated in the same conditions (Figures 1(a) and 1(b)).

**Figure 2:**
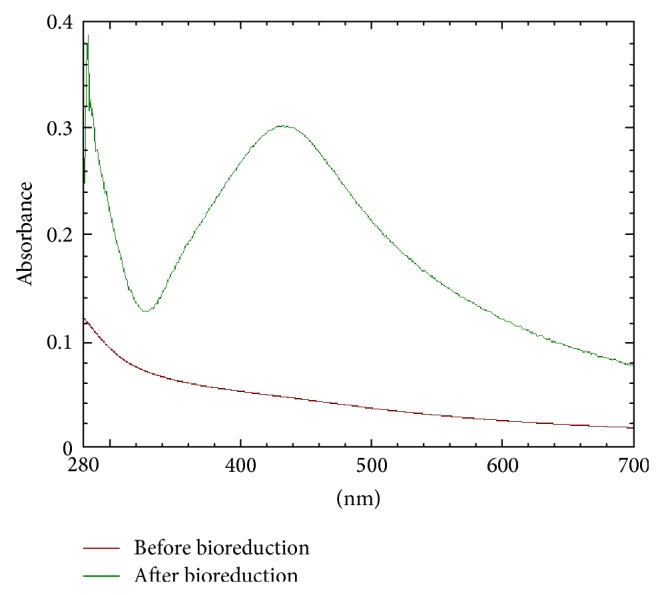
The UV/Vis absorption spectra of silver nanoparticles. nm: wavelength in nanometers (1 unit = 100 nm); Abs: absorbance (1 unit = 0.1 absorbance unit).

The UV/Vis absorption spectrum [21] resulted in the formation of silver nanoparticles at the visible range of 420 nm and this may be due to the fact that both the conduction and valence bands would have lain very close to each other permitting the free flow of electrons that might have subsequently given rise to the surface plasmon resonance (SPR) absorption band as evidenced by Taleb et al. [22], Link and El-Sayed [23], and Noginov et al. [19]. Moreover, the long tailing on the large wavelength (Figure 2) could have been due to the small amount of aggregated particles as reported by Maliszewska et al. [12] and proved in the study that the solution containing nanoparticles remained stable for more than four months, with no signs of aggregation or precipitation showing the SPR peak at the same wavelength [24].

### 3.2. SEM Analysis

**Figure 3:**
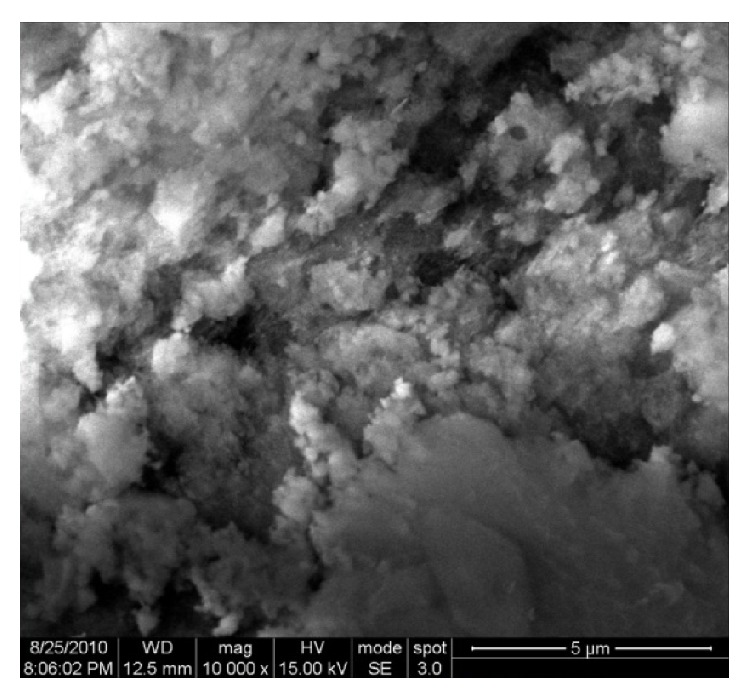
Scanning electron micrograph of silver nanoparticles. WD: working distance between the sample surface and the low portion of the lens which should be greater than 4 mm (12.5 mm); Mag: magnification at 10000x; HV: high voltage (15 kV); Mode: secondary electron (SE) mode.

The SEM micrographs of nanoparticle obtained in the filtrate were magnified up to 10,000x at 15 kV and represented the aggregation of silver nanoparticles, which may be due to the sample preparation (including drying) that could have affected its size and shape as reported by Sadowski et al. [25]. However, from the image the particle size could be confirmed to exist below 5 *μ*m (Figure 3). In fact, the optical absorption spectra of metal nanoparticles shift to longer wavelengths with increasing particle size. According to He et al. [26], small spherical nanoparticles (<20 nm) exhibit a single surface plasmon band. In general, the biosynthesis of silver nanoparticles ranges from 1 to 70 nm and they have different shapes including spherical, triangular, and hexagonal in forms such as bacteria [5, 6], fungi [27–29], plants, and plant extract [4, 15, 30]. Thus, the occurrence of spherical nanoparticles in the present study could be attributed to the evidences made by Raut Rajesh et al. [31], according to whom the single surface plasmon peak obtained at 420 nm even at fourth month could prove the stability of the silver nanoparticles synthesized in the organisms.

### 3.3. Identification of Functional Groups by FTIR

**Figure 4:**
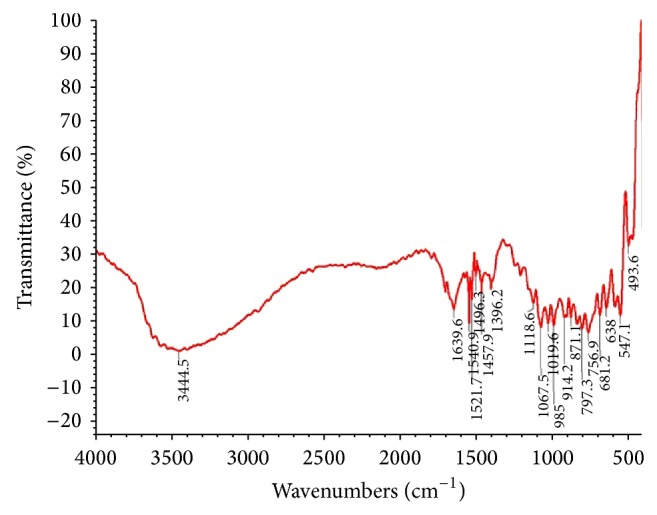
FTIR analysis of synthesized silver nanoparticles from* Westiellopsis *sp. (A.15). Wavenumbers cm^−1^: 1 unit = 500 cm^−1^; % transmittance: 1 unit = 10% transmittance.

FTIR characterization reveals the presence of many different functional groups and earlier reports suggested the role of enzymes or proteins in the synthesis of silver nanoparticles [32, 33]; accordingly, NADH and NADH-dependent nitrate reductase, when induced by nitrate ions, subsequently reduce silver ions to metallic silver by the electron shuttle enzymatic metal reduction process as observed in the fungus* Fusarium oxysporum*. Since* Westiellopsis* sp. (A15) is also known to secrete the cofactor NADH and NADH-dependent nitrate reductase enzyme, this might be responsible for the bioreduction of Ag^+^ to Ag^0^ and subsequently for the formation of silver nanoparticles because the involvement of nitrate reductase in the production of silver nanoparticles in vitro has already been demonstrated by Kumar et al. [34]. Moreover, in certain cyanobacterial species, several naphthoquinones and anthraquinones with excellent redox properties are reported to be involved in the electron shuttle for metal reduction [35, 36], besides the other molecules like polyol, water-soluble heterocyclic components, and chlorophyll which also influence the reduction of silver ions and capping of silver nanoparticles, respectively [30, 37]. Hence, FTIR measurements were carried out to identify the possible biomolecule responsible for the reduction of Ag^+^ ions and capping of the bioreduced silver nanoparticles by cyanobacterial filtrate. Representative spectra of silver nanoparticles (Figure 4) manifested absorption peaks located from less than 400 cm to 4000 cm and indicated the specific functional groups. However, the most prominent peaks with important functional groups were located at 1020, 1068, 1119, 1396, 1458, 1496, 1522, 1541, and 1640 peaks, respectively, in the regions between 1,000 and 1,800 cm^−1^ and their corresponding functional groups were identified through the library [17] and were represented in Table 1.

The band at 1020 cm^−1^ corresponded to the functional group C–O; similarly, bands at 1068 and 1119 cm^−1^ corresponded to both CO stretch vibrations and an aliphatic amine CN stretch vibration of the proteins [37, 38]. The absorption peak at 1458 cm^−1^ may be assigned to symmetric stretching vibrations of –COO– (carboxylate ion) groups of amino acid residues with free carboxylate groups in the protein [15] while the peak at 1496 cm^−1^ corresponded to both C–C stretching vibrations of aromatics and asymmetric stretching vibrations of N–O [30, 39]. The two absorption peaks located at around 1522 and 1541 cm^−1^ were assigned to represent amide II of proteins [39] whereas the absorption peak of 1640 cm^−1^ represented NH bend and the stretching vibrations of –C=C– [37]. Moreover, the band at 1396 cm^−1^ was notably accounted for NO^3−^ as reported by Luo et al. [40]. The signatures of infrared regions in the electromagnetic spectrum revealed the presence of different functional groups like C–N, C–O–O, amide linkages, and –COO that correspond to the formation of amino acid residues and those proteins that bind to nanoparticles as reported by Gole et al. [41], according to whom the free amine groups or cysteine residues in the proteins electrostatically attract the negatively charged carboxylate groups that are present in the extract and therefore contribute to the stabilized proteins. Similarly, functional groups such as –C–O–O–, –C–O–, and –C=C– derived from the heterocyclic compounds (“like proteins”) of the cyanobacterial extract could have aided in the capping of ligands to the nanoparticles as reported by Sastry et al. [30] and Sanghi and Verma [38]. Thus, FTIR observation provided an insight into the information that certainly a number of other bioorganic compounds existing in the solution could have participated in the reduction of silver ions and in the stabilization of the nanoparticles formed.

## 4. Conclusion

The biosynthesis of silver nanoparticles has been demonstrated by reaction of AgNO_3_ to the cyanobacterial filtrate at 27°C, where the size of the silver nanoparticle was revealed to be less than 20 nm on the basis of UV absorption peak. However, the SEM image does not show the clear size and the reason for this could be attributed to error in sample preparation. The bioreduction of AgNO_3_ could be associated with the released protein from the cyanobacterium into the filtrate as confirmed by FTIR peaks.

## Figures and Tables

**Table 1:** FTIR peaks and their representative functional groups.

S. number	FTIR peak	Assigned functional groups	References
1	1020	Stretch vibration of C–O	[21]
2	1068, 1119	Stretch vibration of C–O and C–N stretching vibrations of aliphatic amines	[21, 22]
3	1396	Residual NO^3−^	[25]
4	1458	Symmetric stretching vibrations of –COO	[15]
5	1496	C–C stretching vibrations of aromatics and asymmetric stretching vibrations of N–O	[23, 24]
6	1522, 1541	Amide II	[24]
7	1640	N–H bend and stretching vibrations of –C=C–	[21]
